# Injection locking at 2f of spin torque oscillators under influence of thermal noise

**DOI:** 10.1038/s41598-017-18969-5

**Published:** 2018-01-29

**Authors:** M. Tortarolo, B. Lacoste, J. Hem, C. Dieudonné, M.-C. Cyrille, J. A. Katine, D. Mauri, A. Zeltser, L. D. Buda-Prejbeanu, U. Ebels

**Affiliations:** 1 Centro Atómico Constituyentes CNEA, 1650 San Martín and Consejo Nacional de Investigaciones Científicas y Técnicas, C1033AAJ Buenos Aires, Argentina; 2 Univ. Grenoble Alpes, CEA, CNRS, Grenoble INP, INAC, SPINTEC, F38000 Grenoble, France; 3 Univ. Grenoble Alpes, CEA-LETI MINATEC-CAMPUS, 38000 Grenoble, France; 4 HGST, 3403 Yerba Buena Road, San Jose, California 95135 USA; 5 Present Address: International Iberian Nanotechnology Laboratory, Braga, Portugal

## Abstract

Integration of Spin Torque Nano-Oscillators STNO’s in conventional microwave circuits means that the devices have to meet certain specifications. One of the most important criteria is the phase noise, being the key parameter to evaluate the performance and define possible applications. Phase locking several oscillators together has been suggested as a possible means to decrease phase noise and consequently, the linewidth. In this work we present experiments, numerical simulations and an analytic model to describe the effects of thermal noise in the injection locking of a tunnel junction based STNO. The analytics show the relation of the intrinsic parameters of the STNO with the phase noise level, opening the path to tailor the spectral characteristics by the magnetic configuration. Experiments and simulations demonstrate that in the in-plane magnetized structure, while the frequency is locked, much higher reference currents are needed to reduce the noise by phase locking. Moreover, our analysis shows that it is possible to control the phase noise by the reference microwave current (I_RF_) and that it can be further reduced by increasing the bias current (I_DC_) of the oscillator, keeping the reference current in feasible limits for applications.

## Introduction

The increasing demand on miniaturization and the necessity of implementing more and more frequency standards on a single device (GPS, mobile phone, wifi) require new concepts to cover large frequency ranges at the nanoscale size, keeping costs low. In this sense the Spin STNOs where a spin polarized current passing through the magnetic multi-layered nanosystem can drive the magnetization into large amplitude periodic oscillations^1–3^ when the spin polarized current is large enough to compensate the natural damping, are an alternative path to current controlled microwave devices. Despite all their appealing features, as nano size, fast frequency tuning, high modulation speed and CMOS compatibility, one of the main issues that remains to be addressed is their low phase stability leading to a large phase noise^4–9^, which is the dominant contribution to linewidth in oscillators. Several options were proposed to stabilize the phase: electric coupling^10,11^, dipolar coupling^12–15^ or spin wave coupled nanocontacts^16,17^. In order to understand the role of phase noise in electric synchronization of several oscillators by their own emitted microwave current, we studied the injection locking of an STNO to a reference microwave current I_RF_, with known spectral specifications. Here we focus on standard uniform in plane magnetized oscillators (in-plane polarizer and in-plane free layer, IP), for which an in-plane precession (IPP) mode is stabilized. The injection locking of such an STNO to a reference current at two times the generated frequency (f_ext_ = 2f_o_) was demonstrated both numerically and by experiments^18^. However, the linewidth in the locked regime was reduced only by a factor of seven, while a reduction to the linewidth of the microwave source (several Hz) was expected. These large linewidths are associated to the thermal noise that induces fluctuations which can drive the phase from an equilibrium state to a neighbouring one, with an associated phase slip of ±2π which can be envisaged as non-syncronization and re-synchronization events. Zhou *et al*.^19^ demonstrated that the particular way the phase approaches its synchronized value has consequences in the transients that may limit the modulation of an STNO. Recent works investigated the mechanisms of the so called pure phase locking state in double vortex based STNO: Robust synchronization was experimentally shown, with a 10^5^ linewidth reduction^20^ and the role of the phase slips in the synchronized state was investigated^21^. In this work we study the injection locking at *2 f* to an external reference current of a uniform IP magnetized STNO under the influence of thermal noise. Contrary to most studies in injection locking, here the phase noise is investigated in detail to understand the non ideal locking reported on uniform IP magnetized STNO’s^18^, providing an analytical expression that relates the phase noise level with the intrinsic parameters of the STNO.

## Analytic model

The effect of thermal fluctuations on the transient behaviour of the injection locking state of an STNO is analyzed in the frame of a generic model of a non-linear auto oscillator^21^ that we extended for the IPP mode synchronized by a reference microwave current at 2 *f* (details in Supplementary material). Since STNO’s are non-linear (non-isochronous) oscillators, the power and the phase of the oscillator are not independent, leading to a system of coupled equations.

1 $$\frac{d\psi }{dt}=-{\rm{\Delta }}\omega +2N\delta p+\frac{1}{\sqrt{{p}_{o}}}\text{Im}[\xi ]$$

2 $$\frac{d\delta p}{dt}\cong \,\,-2{{\rm{\Gamma }}}_{p}\delta p\,+2{p}_{0}F\,\cos (\psi )+2\sqrt{{p}_{o}}\mathrm{Re}[\xi ]$$

Here ψ(t) = 2Φ − ω_ext·_t is the phase difference between the STNO phase Φ and the phase of external source ω_ext·_t, *N* is the coefficient of non-linear frequency shift, *F* is a real parameter proportional to the reference current, Γ_p_ is the damping rate of the power fluctuations and ξ has the statistical properties of the Gaussian thermal noise (<ξ(t)> = 0, <ξ(t)ξ(t)> = 0, <ξ(t)ξ(t)*> = δ(t − t′)). Linearizing the equations (1) and (2) around a stable solutions p_o_ and ψ_ο_ (without considering thermal noise) allows us to study the transient behaviour of the synchronized state, finding two solutions for the decay rate λ:

3 $${\lambda }_{1,2}={{\rm{\Gamma }}}_{p}[1\pm \sqrt{1-\frac{\varepsilon }{{\varepsilon }_{c}}}]$$

Here, ε = I_RF_/I_DC_ and ε_c_ = Γ_p_^2^/(2Np_0_^2^sinψ_s_P_x_Γ_J_|*B*|/*A*) (see Supplementary material for the definition of the parameters). When ε > ε_c_, λ is complex with a real part given by Γ_p_, that is the decay rate to the phase locked state and an imaginary part that describes an oscillatory approach to the phase-locked state with a frequency given by:

4 $${{\rm{\Omega }}}_{t}={{\rm{\Gamma }}}_{p}\sqrt{\varepsilon /{\varepsilon }_{c}-1}$$

**Figure 1:**
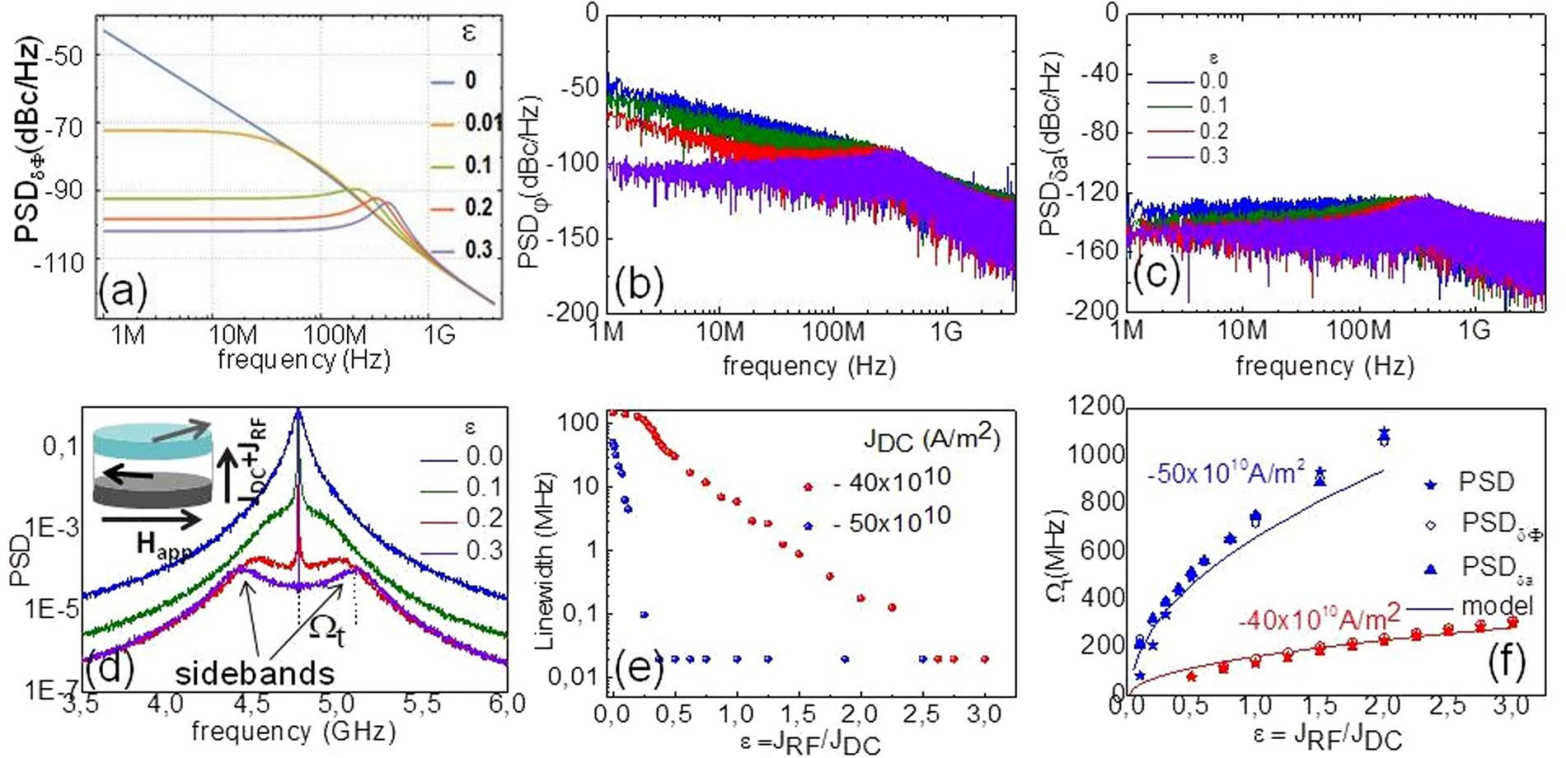
Phase noise calculated from the analytic model (**a**) and numerical simulations at 50 K for the phase (**d**) and amplitude (**c**) noise. Notice the peaks appearing ~200–300 MHz. (**d**) PSD of the signal as a function of ε for J_DC_ = −50 × 10^10^ A/m^2^ and schematics of the oscillator (inset). (**e**) Linewidth vs. ε for low current J_DC_ = −40 × 10^10^ A/m^2^ (red dots) and medium current J_DC_ = −50 × 10^10^ A/m^2^ (blue dots). For ε > 0.3 (medium J_DC_ regime), the linewidth falls below the resolution of the technique.(f) Sideband frequency for both low current regime (red set) and medium current regime (blue set) extracted from the PSD (stars), and from the peaks on the phase noise (Fig. 1b) (open circle). The solid line corresponds to the analytical model developed for the IPP geometry (eq. 4).

This is in agreement with Zhou *et al*.^19^, where they found for out of plane (OP) magnetized STNO’s that the phase approaches its locked state exponentially and oscillating above a certain critical reference current. These oscillations lead to sidebands of frequency Ω_t_ at both sides of the emission peak of the STNO as shown in Fig. 1d.

Before discussing in more detail the oscillatory transient, we first will provide an expression for the phase noise in the synchronized state. By taking into account the thermal noise, and supposing that phase and power are small deviation from the equilibrium we can calculate from eqs 1, 2 the power spectral density (or single sideband) *S*_*δΦ*_ of the phase fluctuations of the synchronized state:

5 $${S}_{\delta \Phi }=2\pi {\rm{\Delta }}{f}_{o}\frac{{(\frac{{{\rm{\Gamma }}}_{p}}{\pi })}^{2}(1+{\nu }^{2})+{f}^{2}}{{[-{(\frac{{{\rm{\Gamma }}}_{p}}{2\pi })}^{2}\frac{\varepsilon }{{\varepsilon }_{c}}+{f}^{2}]}^{2}+4{{\rm{\Gamma }}}_{p}^{2}{f}^{2}}$$

Here ν = Np_o_/Γ_p_ is the dimensionless nonlinear frequency shift and Δf_o_ is the free running linear linewidth. Eq. 5 is plotted in Fig. 1a with the parameters calculated from the analytical model (see Supplementary Material) for the free running state with a bias current J_DC_ = 50 × 10^10^A/m^2^, which leads to an IPP stable precession mode around 4.7 GHz and a Δf_o_ = 50 MHz. In this configuration the system has a coefficient of nonlinear frequency shift N = −3.16·10^10^ rad/sec, a damping rate of the power fluctuations Γ_p_ = 666 Mrad/sec, a normalized dimensionless nonlinear frequency shift parameter |ν| = 16, and ε_c_ = 0.025. Since this value of ε_c_ for these uniform IP STNO’s is small compared to ε (~0.1 or higher) the phase locking always takes place via an oscillatory transient.

The characterization of the phase noise properties by the PSD in the Fourier space has the advantage that its inverse power law dependence on frequency PSD ~1/f ^x^ provides information about underlying noise processes. The model predicts a crossover from 1/f^2^ to 1/f° with increasing reference current, with the two limit cases:

6 $${S}_{\delta \Phi }=\{\begin{array}{c}f\gg {f}_{roll-off}:2\pi {\rm{\Delta }}{f}_{o}(\frac{1}{{f}^{2}+4{{\rm{\Gamma }}}_{p}^{2}})\\ f\ll {f}_{roll-off}:2\pi {\rm{\Delta }}{f}_{0}\frac{4(1+{\nu }^{2})}{{({{\rm{\Gamma }}}_{p}/\pi )}^{2}}{(\frac{{\varepsilon }_{c}}{\varepsilon })}^{2}\end{array}$$

As already shown experimentally^6^ for IPP STNO’s, the free running oscillator (ε = 0) shows a 1/f^2^ dependence associated to a random walk of the phase (blue line, Fig. 1a). This behaviour is modified when applying a reference current I_RF_ at 2f: Even for a low external force (ε = 0.1, yellow line, Fig. 1a) below the roll of frequency f_roll off_ ~1/Γ_p_ down to the lowest (calculated) offset frequency the phase noise is constant. This corresponds to fluctuations of the phase around its locked value. The Eq. (6) shows that the phase noise level in this region can be decreased upon increasing the reference current ε. Nevertheless the phase noise falls as 1/ε^2^ hence for higher reference currents the noise is reduced, but at a much smaller rate for further increasing. The lowest achievable phase noise is limited firstly by the STNO’s voltage breakdown and secondly because a large reference current goes beyond the injection locking assumption, which is considered as a weak perturbation. Also the intrinsic parameters of the STNO as f_o_, Γ_p_ and ν play a role in the lowest noise level suggesting that it can be improved by stack engineering. Last, above ε = 0.1 there is a peak around f_roll off_ that is related with the oscillatory relaxation mechanism^19,22–24^, as will be discussed in the next section.

### Macrospin Analysis

The *S*_*δΦ*_ at 50 K extracted from the numerical time integration of the LLG equation is shown in Fig. 1b. The corresponding evolution of the power spectral density of the *my* component of the magnetization with ε and its linewidth are displayed in Fig. 1d and e respectively. Both phase and amplitude noise (Fig. 1b and c) decrease with the reference current and a clear crossover from a 1/f^2^ (random walk) to a 1/f^0^ (white noise) is seen on the phase noise upon increasing ε.

Before addressing the phase noise level in comparison to the analytic results and the linewidth, we now discuss the PSD of the *m*_*y*_ component of the magnetization, Fig. 1d. The peak of the free running state becomes very narrow and two symmetric sidebands appear upon increasing the reference current. These sidebands are also visible on *S*_*δΦ*_ extracted from the simulations (Fig. 1b), that shows a peak around *f*_*roll–off*_ whose frequency depends on ε. Moreover, *S*_*δΦ*_ calculated with the parameters listed above (eq. 5, Fig. 1a) also shows the peaks associated to the sideband frequencies. Figure 1f shows the frequency of these sidebands extracted from the PSD of the *m*_*y*_ component of the magnetization and from the numeric *S*_*δΦ*_ for two different bias currents J_DC_ = −40 × 10^10^ A/m^2^ (red symbols) and J_DC_ = −50 × 10^10^ A/m^2^ (blue symbols). The full line represents the model (eq. 4) for both bias currents. This comparison confirms that the peaks of the phase noise and the sidebands have the same physical origin arising from the oscillatory approach of the transient state. Furthermore the comparison supports the analytic model.

**Figure 2:**
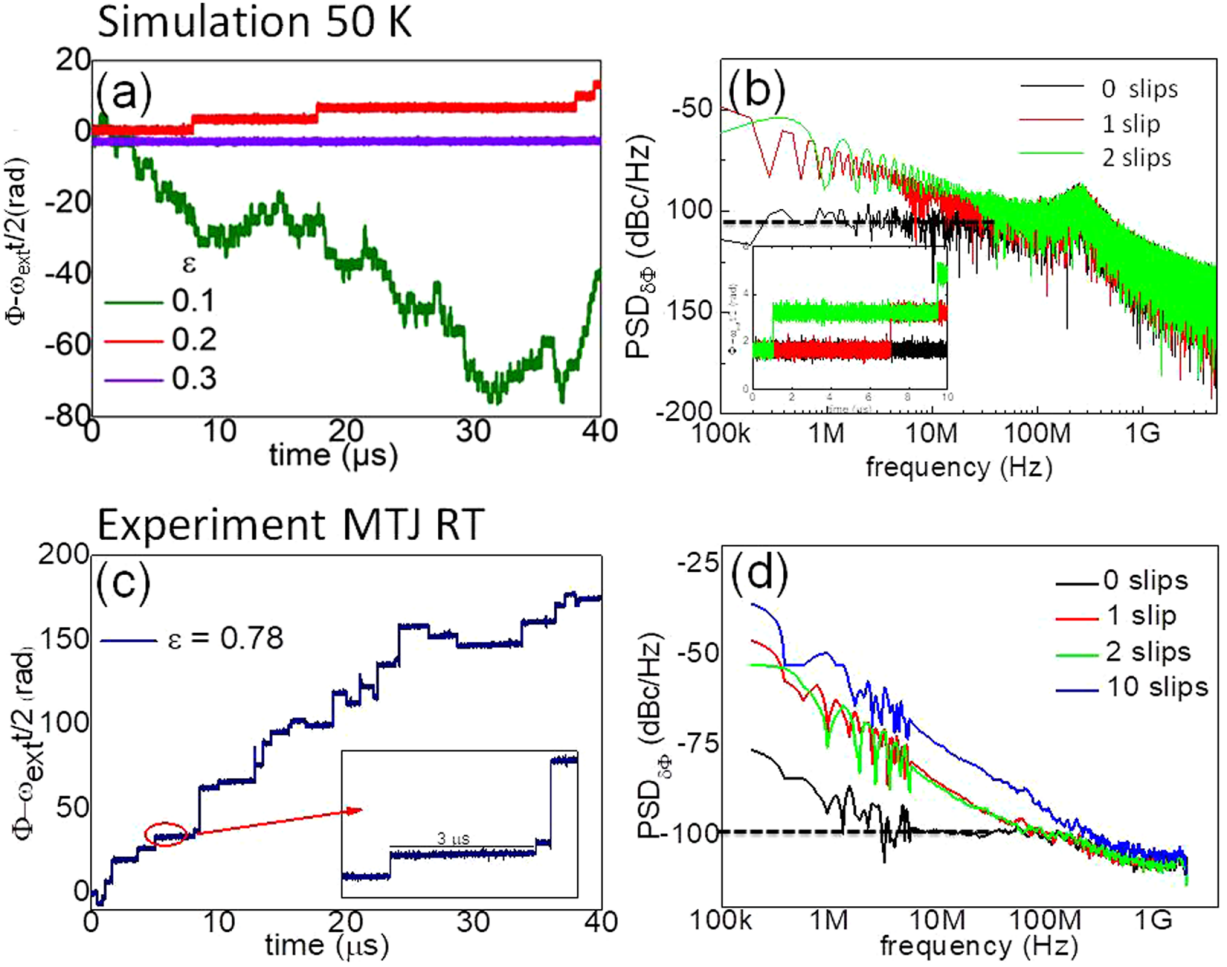
Simulated (**a**) and experimental (**c**) phase temporal traces. Inset: detail of 5μs segments of the temporal trace. The phase slips decrease in number with increasing ε disappearing at ε = 0.3. (**b**) Phase noise analysis on the simulated temporal trace segments (inset) corresponding to no phase slips (black), 1 phase slip (red) and 2 phase slips (green). (**d**) Phase noise analysis of the experimental time trace from 3 s segments with 0, 1, 2, 10 phase slips.

In the following we discuss the phase noise level of the numerical results that show two contributions to the locked state. The first one, as it was discussed for the analytical description, are phase fluctuations around the stable phase which is given by the external source plus a constant phase shift. The second contribution comes from the phase slips^25^, not considered in the analytical model but that are present in the numerical calculation. To understand their contribution to the phase noise and linewidth we extracted the phase from simulated time traces for different reference current values (Fig. 2a). The phase trace shows a drift in time, together with the appearance of the phase slips, which become well defined upon increasing the reference current. As can be seen the number of phase slips per sampling period (~40 μs) decreases with increasing ε. These phase slips are responsible for the 1/f^2^ contribution of the phase noise at low offset frequencies. To demonstrate this, we compare the phase noise extracted from different 10 μs segments of the total 40 µs phase trace that contain respectively none, one or two phase slips. In Fig. 2b it is clearly seen that in presence of phase slips the phase noise has a 1/f^2^ dependence at low frequency f < f_*roll off*_, while in absence of phase slips there is a constant phase noise level of around −100 dBc/Hz.

From the numerical analysis we can see that the drastic decrease in the linewidth with ε (Fig. 1e) can be related to the decreasing number of phase slips. Particularly, when the phase noise flattens for ε > 0.3 (ε > 2.75) for J_DC_ = −50 × 10^10^ A/m^2^ (J_DC_ = −40 × 10^10^ A/m^2^), the phase slips are absent in the phase trace and the linewidth falls under the resolution limit of the numerical calculation (20 kHz). This fact shows that the so called “pure” synchronization^20,26^ is due the absence of phase slips, where the STNO would reduce its linewidth ideally to the one of the reference source. In the case discussed here, this means that for values larger than ε = 0.3 (ε = 2.75) torques from the reference current on the magnetization are strong enough to stabilize the phase around a single value and the remaining noise is given by the one discussed within the analytical model, describing damped oscillations around the stable phase, for noise frequencies f < f_*roll off*_. We point out here that the absence of the phase slips depends on the observation time, i.e. the length of the temporal trace: longer observation times increase the probability of phase slips. These results evidence that even if the system is in the frequency locking regime, higher values of reference current are needed to achieve full linewidth reduction by phase locking. This observation is in agreement with Lebrun *et al*.^26^ that highlighted the difference between the reported “frequency locked state^18,27^” and “pure phase locked state” in absence of phase slips for vortex oscillators with a free running frequency of ~200 MHz and ~100 kHz linewidth (free running).

### Experiment

**Figure 3:**
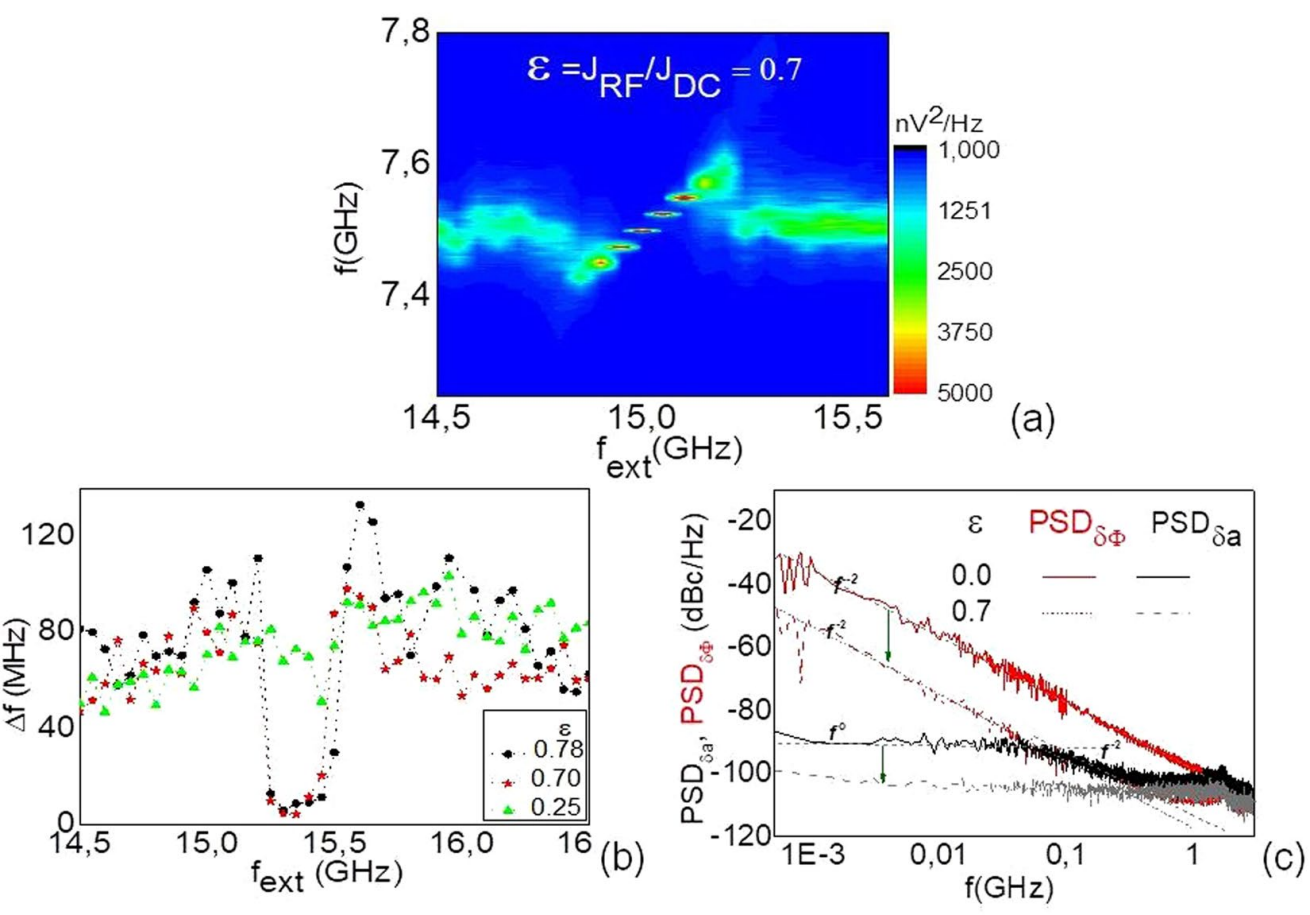
PSD map of the output power at I_RF_ = 1.12 mA (**a**). Linewidth vs ε = I_RF_/I_DC_ (**b**) and amplitude and phase noise from the experiment (**c**) for the non-locked state, (continuous line, ε = 0) and locked state (dashed line, ε = 0.7). Notice that the injection locking mechanism is efficient to reduce the noise level (green arrows).

The analytical and numerical results explain qualitatively the experimental observations on the injection locking by a reference current. We present here results for a device with an autonomous, i.e. free running regime characterized by a free running frequency of f_0_ = 7.5 GHz for a bias current I_DC_ = −1.6 mA and an applied in plane field of 350 Oe, with a linewidth of 55 MHz. The PSD map of the output power for the STNO frequency f as a function of the source frequency f_ext_ is shown in the Fig. 3a for ε = J_RF_ /J_DC_ = 0.7. In Fig. 3b it is clearly seen that for increasing reference current ε the linewidth reaches a 10 × reduction (8 MHz with a 1 MHz resolution bandwidth). The amplitude noise shows a 1/f^0^ behaviour both for locking (Fig. 3c, grey dashed line) and the free running state (Fig. 3c, black full line), characteristic of white noise fluctuations of the amplitude around its stable value. The experimental plots of the phase noise in the locking (Fig. 3c, red dashed line) and the free running state (red full line) show that the injection locking mechanism is efficient to reduce the phase noise by 20 dBc/Hz with respect to the non-locked state. Both phase noise plots display a 1/f^2^ behaviour, however the origin is different. In the free running state it results from a random walk of the phase, while in the locking state it is due to the phase slips as explained in the macrospin analysis. Our experiments show that for the maximum applied reference current, even though the oscillator is synchronized with the external source, the emission linewidth remains broad. The phase noise decreases but it does not reach the constant level for which the linewidth is expected to reduce to the source noise. This was not observed in our experiments because the voltage breakdown of the samples did not allow to continue increasing the reference current preventing the STNO from achieving a pure phase locked state. For the same reason, we were not able to observe sidebands in the experimental voltage output. Nevertheless, we have extracted the phase noise from shorter 3 μs segments of the temporal trace^28^ of the phase (Fig. 2c), that include either 0, 1, 2 or 10 phase slips. As can be seen in absence of phase slips the phase noise is flat in a certain range of offset frequencies ~5–100 MHz in Fig. 2d. This demonstrates the lowest phase noise level that can be reached for the in-plane STNO, when phase slips would be completely suppressed. Note that this level of −100 dBc/Hz is the same for the analytic model (Fig. 1a) and simulations (Fig. 1b).

The large free running linewidth of ~50 MHz of our STNO’s could be a drawback to linewidth reduction as discussed by Hamadeth *et al*.^20^ where by decreasing 7 times the free running linewidth, the linewidth reduction in the locked regime goes from only 10 to 10^5^. Indeed, this is also witnessed in our numerical simulations, where the smaller free running linewidth at higher bias current (J_DC_ = −50 × 10^10^A/m^2^) leads to a linewidth reduction for significantly lower reference currents.

### Perspectives

We have studied the synchronization mechanism of a uniform IP magnetized STNO under thermal noise. The synchronization of these devices was demonstrated in several experiments, however no more than a 10 times reduction of linewidth was achieved. This is explained by numerical simulations including thermal noise. While the STNO can be synchronized by moderate reference currents, higher reference currents are needed for full linewidth reduction. With increasing reference current the number of phase slips is reduced resulting in a crossover from 1/f^2^ to 1/f^0^ behaviour in the phase noise when the phase slips are suppressed. The simulations also show that it is possible to achieve linewidth reduction for lower reference currents by increasing the bias current of the oscillator. This study shows the role of the intrinsic parameters of an STNO on the phase noise level, which will be relevant for the design of STNO configurations of appropriate performances for microwave applications in the gigahertz range.

## Methods

We performed macrospin simulations for the in-plane precession (IPP) mode of an in-plane magnetized polarizer and free layer magnetic tunnel junction MTJ, using a solver for the Landau-Lifshitz-Gilbert equation and taking into account the damping like torque term (the field like term was neglected in this work, see Supplementary material). The simulation parameters are the following: free layer of size 90 × 80 × 3.9 nm^3^; spontaneous magnetization M_s_ = 1000 kA/m, damping parameter α = 0.02 and zero magneto-crystalline anisotropy. The polarizer is aligned in the plane at 165° from the free layer magnetization equilibrium position and a spin-polarization η = 0.37 is supposed. A static magnetic field of 40 mT was applied along the in plane easy axis (Ox). The continuous current density was set to J_DC_ = −50·10^10^ A/m^2^, leading to an IPP stable precession mode with f ~ 4.7 GHz. A white Gaussian thermal noise field was added, corresponding to 10 K, 20 K, 50 K, and 100 K^18^. The frequency of the RF current was set to two times the free running STNO frequency 2f_o_ = 9,5396 GHz, which corresponds to the centre of the locking range. The phase and amplitude noise in the synchronized state as a function of the reference current were extracted from the simulated temporal traces of the m_y_ component of the magnetization (in-plane magnetization along the short axis of the pillar) using the Hilbert transform method^29,30^ which allows the reconstruction of an analytic signal from the voltage output:

$$V={V}_{0}(1+\delta a)\cos (2\pi ft+\delta \Phi )$$

The experimental studies were realized on the same type of devices presented in refs^6,18,31^, which are in plane magnetized MTJ, having a stack composition of IrMn/CoFeB/Ru/CoFeB/MgO/CoFe/CoFeB and nominal resistance area product RA = 1 Ωμm^2^. The synchronization experiment was done varying the reference current frequency around two times the free running frequency (2f_o_) of the oscillator, from 14 GHz to 16 GHz, and the source power was varied from −15 dBm to −5 dBm (corresponding to a reference current of ~0.3 to 1.3 mA), just before the sample starts to show signs of degradation. A detailed description of the experiment is available in ref.^18^. The temporal traces were measured using a single shot oscilloscope^4,18,32^, and amplitude and phase noise were extracted using the same protocol as for the simulated data.

## Electronic supplementary material

Supplementary Information
