# Supplementary material for: Injection locking at 2f of spin torque oscillators under influence of thermal noise
Source: Sci Rep. 2018 Jan 29;8:1728. doi: 10.1038/s41598-017-18969-5 (PMC5789033; doi:10.1038/s41598-017-18969-5)
Supplement: Supplementary file 1 — Supplementary Information [file 41598_2017_18969_MOESM1_ESM.pdf]

Injection locking at 2f of spin torque oscillators under influence of thermal noise

M. Tortarolo[1*], B. Lacoste[1**], J. Hem[1], C. Dieudonné[1], M.-C. Cyrille[2], J. A. Katine[3], D. Mauri[3], A. Zeltser[3], L.D. Buda-Prejbeanu[1], U. Ebels[1]

[1]Univ. Grenoble Alpes, CEA, CNRS, Grenoble INP, INAC,SPINTEC, F38000 Grenoble, France
[2]Univ. Grenoble Alpes, CEA-LETI MINATEC-CAMPUS, 38000 Grenoble, France
[3]HGST, 3403 Yerba Buena Road, San Jose, California 95135, USA

Supplementary Material: Model for 2f synchronization in IPP mode

The transient behavior in the synchronized state of a STNO is analyzed in the frame of a generic model of a nonlinear auto-oscillator. The model proposed by ref. [4] is extended for the synchronization by an RF spin-current at 2f. The configuration selected here has both the free-layer and the polarizer in-plane magnetized. The magnetization of the free layer is supposed to be uniform, thus the Gibbs free energy associated of the ferromagnetic free layer of the nanopillar is:

$$E(\mathbf{m}) = K_u\left(1-m_x^2\right)V - \mu_0 M_s H_0 m_x V + \frac{1}{2}\mu_0 M_s^2\left(N_X m_x^2 + N_Y m_y^2 + N_Z m_z^2\right)V \qquad \text{(A1)}$$

Where **m**=**M**/M_s is unitary magnetization vector, V the volume of the sample, M_s the spontaneous magnetization. A static magnetic field of amplitude H_0 is applied in the plane of the sample along the Ox direction. The demagnetizing effects are accounted by the demagnetizing tensor **N**=(N_X, N_Y, N_Z) and an uniaxial magneto-crystalline anisotropy along O with a value K_u (K_u>0). Following the Holstein-Primakoff transformation the variables m_x, m_y, m_z are replaced by the canonical variables (a, a*) such as:

$$a = \frac{m_y - jm_z}{\sqrt{2(1+m_x)}} \qquad \text{(A2)}$$

It is convenient to express the reduced Gibbs free energy in reduced units:

$$\frac{\gamma_0'}{\mu_0 M_s V} E\,(a,a^*) = \mathcal{A}aa^* + \frac{1}{2}\mathcal{B}\left(a^2 + a^{*2}\right) + \mathcal{V}\left(aa^{*3} + a^3 a^*\right) + \mathcal{U}a^2 a^{*2} \qquad \text{(A3)}$$

Where $\gamma_0' = \frac{\mu_0\gamma}{1+\alpha^2}$ with $\alpha$is the damping constant, $\gamma_0 = \mu_0\gamma$ is the gyromagnetic ratio of the free electron multiplied by the vacuum permeability μ_0.The notations are similar to that of Ref. 4.

$$\begin{aligned}
&\mathcal{A} = \omega_A + \omega_H + \omega_M / 2 \\
&\mathcal{B} = -\omega_M / 2 \\
&\mathcal{U} = -(\omega_A + \omega_M / 2) \\
&\mathcal{V} = \omega_M / 4 \\
&\omega_M = \gamma_0' M_S (N_Z - N_Y) \\
&\omega_H = \gamma_0' H_0 \\
&\omega_A = \gamma_0' \left[\frac{2K_u}{\mu_0 M_s} + M_s (N_Y - N_X) \right]
\end{aligned} \qquad \text{(A4)}$$

A second transform is used for the diagonalization of the quadratic part of the reduced Gibbs free energy (Hamiltonian): $b=ua+va^*$ where

$$u = \sqrt{\frac{\mathcal{A}+\omega_0}{2\omega_0}}, v = -\sqrt{\frac{\mathcal{A}-\omega_0}{2\omega_0}} \qquad \text{(A5)}$$

with $\omega_0 = \sqrt{\mathcal{A}^2 - \mathcal{B}^2}$. The last transformation is simply a normalization: $b = \sqrt{\frac{\omega_0}{\mathcal{A}}} c$.

Once a spin-polarized current of polarization **p**=(P_x,P_y, 0) is injected in the sample the modified Gilbert equation is used to describe the magnetization dynamics considering the damping-like term of the spin-transfer:

$$\frac{d\mathbf{m}}{dt} = -\gamma_0 (\mathbf{m} \times \mathbf{H_{eff}}) + \alpha \left(\mathbf{m} \times \frac{d\mathbf{m}}{dt} \right) - \gamma_0 a_J J_{app} \mathbf{m} \times (\mathbf{m} \times \mathbf{p}) \text{(A6)}$$

The effective field is given by the functional derivative of the Gibbs free energy with respect to the magnetization: $\mathbf{H_{eff}} = -\frac{1}{\mu_0 M_s V} \frac{\delta E}{\delta \mathbf{m}}$. The injected current density is time dependent and given by:

$$J_{app}(t) = J_{DC} + J_{RF} \cos(\omega_{ext} t) = J_{DC} [1 + \varepsilon \cos(\omega_{ext} t)] \qquad \text{(A6)}$$

The spin–torque amplitude coefficient is $a_J = \frac{\hbar}{2e} \frac{\eta}{\mu_0 M_s t}$ where t is the thickness of the free layer and η is the spin-polarization.

The numerical analysis is carried out on the equivalent modified Landau-Lifshitz equation:

$$\frac{d\mathbf{m}}{dt} = -\gamma_0' (\mathbf{m} \times \mathbf{H_{eff}}) - \alpha \gamma_0' [\mathbf{m} \times (\mathbf{m} \times \mathbf{H_{eff}})] - \gamma_0' a_J J_{app} \mathbf{m} \times (\mathbf{m} \times \mathbf{p}) + \gamma_0' \alpha a_J J_{app} (\mathbf{m} \times \mathbf{p}) \text{ (A7)}$$

Applying the three transformations presented above $\mathbf{m} \to (a, a^*) \to (b, b^*) \to (c, c^*)$, the full expression of the dc/dt equation contains 25 terms, each of them being proportional with the product between power of c and power of c* :$Kc^m c^{*n} = |c|^{m+n} \exp(-j(m-n)\Phi)$ with m, n integers and K a coefficient with can be time dependent ~$\exp(\omega_e t)$. To find the stationary solution one have to compute the time average over one period. Some of the above mentioned terms (the fast

varying, e.g. m=3n) have a zero average value and thus they are neglected (minor role on the average behavior of the oscillator). Other terms are resonant (e.g. m=3, n=1, K=~ exp(ω_et)) since their average value is not zero over a period and contribute thus to the power in synchronized state. Keeping only the potential resonant terms the following equation for the complex variable *c* is obtained:

$$\frac{dc}{dt} = -j\left[\omega_0 + N|c|^2\right]c - \Gamma_0\left[1 + Q_1|c|^2 + Q_2|c|^4\right]c - \Gamma_J P_x\left[1 + \varepsilon\cos(\omega_e t)\right]\left[1 - |c|^2 - \frac{|\mathcal{B}|}{2\mathcal{A}}\left(c^2 + c^{*2}\right)\right]c$$
$$+ \Gamma_J P_y\left[1 + \varepsilon\cos(\omega_e t)\right]\frac{1}{2}\sqrt{\frac{|\mathcal{B}|}{\omega_0}}(u + v)$$

(A8)

The expressions for the coefficients are the following:

$$N = -\left[3\omega_M uv\left(u^2 + v^2\right) + \left(2\omega_A + \omega_M\right)\left(u^4 + v^4 + 4u^2v^2\right)\right]\frac{\omega_0}{\mathcal{A}}$$
$$\Gamma_0 = \alpha\mathcal{A} \qquad \text{(A9)}$$
$$Q_1 = -\left[3uv\omega_M + \left(u^2 + v^2\right)\left(\omega_H + 3\omega_A + \frac{3}{2}\omega_M\right)\right]\frac{\omega_0}{\mathcal{A}^2}$$
$$Q_2 = \left[3uv\left(u^2 + v^2\right)\omega_M + \left(u^4 + 4u^2v^2 + v^4\right)\left(2\omega_A + \omega_M\right)\right]\left(\frac{\omega_0}{\mathcal{A}}\right)^2$$
$$\Gamma_J = \gamma'_0\, a_J J_{DC}$$

In Eq. A8 it is important to keep the term involving c^2+c^{*2} since it mediates the 2f synchronization. This has been neglected until now in the literature.

The analysis is continued in terms of power *p* and phase Φ such as $c = \sqrt{p}e^{-j\Phi}$ for which two coupled equations are obtained:

$$\begin{cases} \dfrac{dp}{dt} = -2\Gamma_0\left[1 + Q_1 p\right]p - 2P_x\Gamma_J\left[1 + \varepsilon\cos(\omega_e t)\right]\left[1 - p - \dfrac{|\mathcal{B}|}{\mathcal{A}}p\cos(2\Phi)\right]p \\ \qquad + 2P_y\Gamma_J\left[1 + \varepsilon\cos(\omega_e t)\right]\dfrac{1}{2}\sqrt{\dfrac{|\mathcal{B}|}{\omega_0}}(u + v)\sqrt{p}\cos\Phi \\ \dfrac{d\Phi}{dt} = \left[\omega_0 + Np\right] - P_y\Gamma_J\left[1 + \varepsilon\cos(\omega_e t)\right]\dfrac{1}{2}\sqrt{\dfrac{|\mathcal{B}|}{\omega_0}}(u + v)\dfrac{\sin\Phi}{\sqrt{p}} \end{cases} \qquad \text{(A10)}$$

In DC regime (J_{RF}=0) these generic equations allow to extract the power p_0 in the free-running state:

$$p_0 = -\frac{\Gamma_0 + P_x\Gamma_J}{\Gamma_0 Q_1 - P_x\Gamma_J} \qquad \text{(A11)}$$

and to define the amplitude relaxation rate:

$$\Gamma_p = \left(\Gamma_0 Q_1 - P_x\Gamma_J\right)p_0 . \qquad \text{(A12)}$$

In the 2f synchronized state the phase difference between the STNO and the external signal $\psi = 2\Phi - \omega_{ext}t$ is constant and the stationary power p_s is shifted by $\delta p = p_s - p_0$.

Keeping the resonant terms at 2f (non zero time average over an oscillation´s period), it is possible to obtain the coupled equations:

$$\begin{cases} \dfrac{d\psi}{dt} = -\Delta\omega + 2N\delta p \\ \dfrac{d\delta p}{dt} \cong -2\Gamma_p \delta p + 2p_0^2 \mathcal{F}\cos(\psi) \end{cases} \qquad \text{(A13)}$$

with $\Delta\omega = \omega_{ext} - 2(\omega_0 + Np_0)$ and $\mathcal{F} = \varepsilon P_x \Gamma_J \dfrac{|\mathcal{B}|}{2\mathcal{A}} p_0$.

The stable stationary solution corresponding to the 2f synchronized state has the phase difference given by $\psi_s = \arccos\left(\dfrac{\Delta\omega}{\Omega}\right)$ with $\Omega = \dfrac{2Np_0}{\Gamma_p}\mathcal{F}$ and a stationary shift in power given by $\delta p_s = \dfrac{\Delta\omega}{2N}$.
